# Effects of natriuresis-guided diuretic therapy in men and women with acute heart failure: insights from PUSH-AHF

**DOI:** 10.1007/s00392-026-03030-1

**Published:** 2026-09-28

**Authors:** Hailun Qin, Lara E. E. C. Zonneveld, Adriaan A. Voors, Iris E. Beldhuis, Alice Ravera, Dirk J. van Veldhuisen, Jenifer E. Coster, Wybe Nieuwland, Jan A. Krikken, Peter van der Meer, Kevin Damman, Jozine M. ter Maaten

**Affiliations:** 1 Department of Cardiology, University Medical Center Groningen, University of Groningen, Hanzeplein 1, 9713 GZ Groningen, the Netherlands; 2 Institute of Cardiology and Department of Medical and Surgical Specialties, Radiologic Sciences and Public Health, University of Brescia, ASST Spedali Civili Di Brescia, Brescia, Italy

**Keywords:** Acute heart failure, Natriuresis, Natriuretic response, Sex, Worsening renal function

## Abstract

**Aim:**

Natriuresis-guided therapy has been proposed as a personalized approach for decongestion in acute heart failure (HF). Given the evidence of sex-related differences in response to decongestive therapy, we performed a sex-specific analysis on the treatment effect of natriuresis-guided therapy in the PUSH-AHF trial.

**Methods:**

The PUSH-AHF trial randomized 310 patients (45% women) to natriuresis-guided therapy or standard of care (SOC). The endpoints of PUSH-AHF were compared between men and women. The 24-h natriuretic response was calculated as 24-h natriuresis divided by the total 24-h loop diuretic dose (mg of bumetanide equivalents).

**Results:**

In men, mean total 24-h natriuresis was greater with natriuresis-guided therapy compared to SOC (443 vs. 354 mmol, *p* = 0.004), whereas in women, this was similar between the groups (357 vs. 336 mmol, *p* = 0.533, adjusted *p*-interaction = 0.276). The median 24-h natriuretic response was lower in women receiving natriuresis-guided therapy (23 vs. 52 mmol urinary sodium/mg bumetanide, *p* = 0.001) when compared to SOC, whereas in men, there was no significant difference between treatment arms (38 vs. 45 mmol respectively, *p* = 0.337, adjusted *p*-interaction = 0.009). Natriuresis-guided therapy was associated with a lower risk of 180-day HF rehospitalization or all-cause mortality in men (adjusted HR 0.51 (95% CI, 0.30–0.86; *p* = 0.011), but not in women (adjusted HR 1.40 (95% CI, 0.67–2.91; *p* = 0.368), adjusted *p*-interaction = 0.026).

**Conclusion:**

Natriuresis-guided therapy was associated with greater natriuresis and a lower risk of 180-day all-cause mortality or HF rehospitalization in men but not in women. These exploratory sex-specific patterns warrant further investigation and confirmation.

**Graphical Abstract:**

**Figure:**
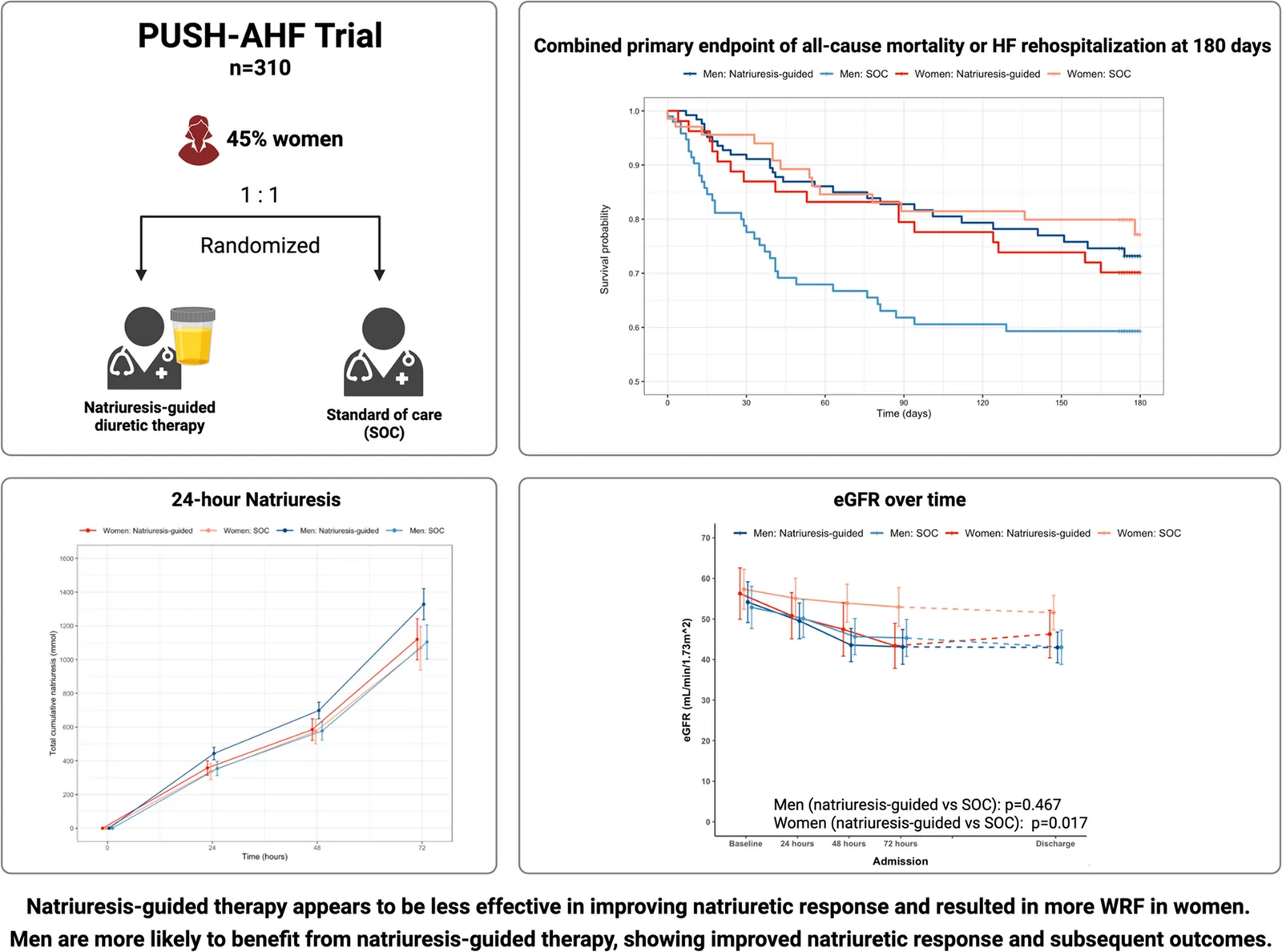

**Supplementary Information:**

The online version contains supplementary material available at https://doi.org/10.1007/s00392-026-03030-1.

## Introduction

Sex differences in chronic heart failure (HF) are well-recognized. Women are typically older, more likely to present with heart failure with preserved ejection fraction (HFpEF), and report poorer quality of life, yet they generally experience better survival compared to men with heart failure [1–3]. In patients with HFpEF, sex-related treatment heterogeneity has been observed, with women appearing to derive greater benefit from sacubitril/valsartan and spironolactone than men [4, 5]. Furthermore, women with heart failure with reduced ejection fraction (HFrEF) may achieve the lowest risk of HF hospitalization or all-cause mortality at around 40% of the target doses for angiotensin-converting enzyme (ACE) inhibitors/angiotensin receptor blockers (ARBs) and 60% for β-blockers, whereas men benefit most from reaching the guideline-recommended target doses [6]. Sex-specific differences during episodes of acute decompensated HF remain poorly understood, particularly regarding response to decongestive therapy. To date, only a few studies have reported sex-specific data on diuretic therapy. The ADHERE [7] study found similar intravenous diuretic regimens in men and women, whereas the ASCEND-HF [8], RELAX-AHF [9], RELAX-AHF-2 [10], and PROTECT [11] trials reported that women received less intensive diuretic therapy during admission, achieved less diuresis, and had lower in-hospital weight loss compared to men, however had earlier improvement in general well-being. These findings suggest potential sex-specific differences in decongestion management and/or response to therapy during acute episodes of HF that warrant further investigation.

The European Society of Cardiology HF guideline recommends early evaluation of diuretic response and dose adjustment of diuretics based on natriuresis and diuresis during hospitalization, [12] highlighting the importance of natriuresis as a key marker to guide decongestive therapy. However, sex-specific differences in response to natriuresis-guided therapy among patients hospitalized with acute HF have not been examined so far. The PUSH-AHF trial provided the first opportunity to investigate sex-specific treatment effect of natriuresis-guided diuretic therapy in patients with acute HF within the context of a randomized controlled trial (RCT). While there was no statistically significant interaction between treatment arm and sex for the first part of the dual primary endpoint, 24-h natriuresis (*p*-interaction = 0.1399), sex appeared to modify the effect of natriuresis-guided diuretic therapy versus standard of care (SOC) on the second part of the dual primary endpoint, namely 180-day all-cause mortality and HF rehospitalization [13]. Given the potential clinical relevance of this finding, we conducted a detailed subgroup analysis to explore the similarities and differences between men and women in the PUSH-AHF trial and to further examine the interaction between the effect of natriuresis-guided therapy and sex.

## Methods

### PUSH-AHF design and patients

PUSH-AHF trial was a prospective, single-center, pragmatic, open-label, RCT that investigated the effects of the natriuresis-guided diuretic therapy versus SOC on natriuresis and clinical outcomes in patients hospitalized with acute HF. The rationale, design, and primary results of the PUSH-AHF trial have been published previously [13, 14]. Briefly, 310 patients who presented with acute HF at the University Medical Center Groningen (UMCG), the Netherlands, were 1:1 randomly assigned to receive either SOC or natriuresis-guided diuretic therapy at the time of initial intravenous diuretic therapy. In both arms, the initial dose of loop diuretic therapy was pre-specified by protocol and based on outpatient loop diuretic use and baseline estimated glomerular filtration rate (eGFR, calculated using Chronic Kidney Disease Epidemiology Collaboration formula). The SOC arm received loop diuretics twice a day. In the natriuresis-guided diuretic therapy arm, spot urinary sodium and urinary volume were measured at predetermined time points (2–6–12–18–24–36 h), and diuretic therapy was intensified if spot natriuresis was less than 70 mmol/L and/or diuresis was less than 150 mL/h with persistent congestion.

The protocol was approved by the Medical Ethics Review Committee of UMCG (METC 2020/587) and was conducted in accordance with the Declaration of Helsinki and the International Conference of Harmonization Guidelines for Good Clinical Practice. All patients provided written informed consent.

### Definition of primary and secondary outcomes

The first 24 h total natriuresis and the combined endpoint of all-cause mortality or first adjudicated HF rehospitalization at 180 days were the dual primary outcomes of the PUSH-AHF trial. Length of hospital stay; all-cause mortality; HF rehospitalization; 48- and 72-h percentage change in N-terminal pro-B-type natriuretic peptide (NT-proBNP); 48- and 72-h natriuresis; and 24-, 48-, and 72-h diuresis were the key secondary outcomes. HFpEF was defined as a left ventricular ejection fraction (LVEF) greater than 50%.

### Definition of natriuretic response and worsening renal function

Total 24-h natriuresis was stratified to good or poor natriuresis based on whether values were above or below the median for the overall population (median, 341 mmol). The 24-h natriuretic response was defined as the total 24-h natriuresis divided by the total administered loop diuretic dose in the first 24 h (mg of bumetanide equivalents). Worsening renal function (WRF) was defined as an absolute increase in serum creatinine ≥ 26.5 µmol/L (0.3 mg/dL), with or without a relative increase in serum creatinine > 25%, or a relative decrease in eGFR > 25% between baseline and 72 h [15].

### Statistical analysis

Patients were stratified by sex and treatment allocation, with baseline characteristics presented separately for men and women. Categorical variables were reported as counts (percentages, %), normally distributed variables as mean (standard deviation, SD), and non-normally distributed variables as median [interquartile ranges, Q1;Q3]. Group differences were assessed using the chi-square test for categorical variables, Student’s *t*-test for normally distributed data, and the Mann–Whitney *U* test for non-normally distributed data, as appropriate. Linear regressions and Cox regression analyses were used to evaluate the associations between treatment arms and endpoints in men and women. The interaction between sex and treatment on primary and secondary endpoints was calculated. Before conducting regression analyses, multicollinearity and the proportional hazards assumption were checked. Regression models were further adjusted for baseline differences between men and women, including heart rate, heart failure etiology, myocardial infarction, and baseline levels of sodium, potassium, and creatinine. No adjustments for multiple testing were performed. The total effect of natriuresis-guided diuretic therapy on changes in eGFR during admission was analyzed using a linear mixed-effects model, adjusted for baseline eGFR, time, and treatment allocation, with a random intercept for patient to account for repeated eGFR measurements. To assess whether eGFR trajectories differed by sex, the treatment-by-time-by-sex interaction was evaluated. All available eGFR measurements during admission were included in the analysis. A statistically significant two-sided *p*-value was defined as less than 0.05. All analyses were conducted using R, version 4.3.2.

## Results

###  Baseline characteristics in men and women

Baseline characteristics for men and women are summarized in Table 1 and Supplemental Tables 1 and 2. In total, 172 men (55%) and 138 women (45%) were randomized. Baseline LVEF was available in 231 patients (75%), of which 18% of men and 27% of women had HFpEF (*p* = 0.137). Men had significantly lower heart rate, more often had ischemic HF, and exhibited slightly higher baseline serum sodium, potassium, and creatinine levels compared to women (all *p* < 0.05) (Table 1). Signs and symptoms of congestion were similar between men and women. Men and women received similar HF background medication and device therapy. Baseline characteristics were well-balanced between natriuresis-guided diuretic therapy and SOC arms in both men and women (Supplemental Tables 1 and 2).

**Table 1:** Baseline characteristics for men and women, overall

	Men ***N*** = 172	Women ***N*** = 138	***p***-value
Treatment arm			0.228
Natriuresis-guided therapy	89 (52%)	61 (44%)	
Standard of care	83 (48%)	77 (56%)	
Demographics			
Age (in years) at time of screening	73 [65;80]	76 [65;84]	0.100
Race, Caucasian	166 (97%)	131 (97%)	0.688
Physical examination			
BMI (kg/m^2^)	28 (5)	29 (7)	0.548
Systolic blood pressure (mmHg)	131 (26)	132 (26)	0.898
Diastolic blood pressure (mmHg)	81 (18)	80 (20)	0.654
Heart rate	89 (25)	99 (30)	0.002
NYHA class			0.336
Class II	9 (5%)	7 (5%)	
Class III	43 (25%)	25 (18%)	
Class IV	120 (70%)	106 (77%)	
HF characteristics			
LVEF (%)	35 [28;54]	35 [25;48]	0.050
HFpEF (LVEF > 50%)	24 (18%)	27 (27%)	0.137
Ischemic etiology	79 (46%)	32 (23%)	< 0.001
New-onset HF	72 (42%)	63 (46%)	0.610
Time since HF diagnosis (months)	8 [0;78]	20 [0;74]	0.804
HF hospitalization within the past year	29 (17%)	12 (9%)	0.052
Medical history			
Myocardial infarction	68 (40%)	32 (23%)	0.003
Hypertension	110 (64%)	85 (62%)	0.757
Atrial fibrillation	96 (56%)	78 (57%)	0.992
Diabetes	68 (40%)	50 (36%)	0.633
Depression	11 (6%)	6 (4%)	0.592
Laboratory values			
Hemoglobin (mmol L^−1^)	7.7 [6.9;8.7]	7.8 [7.0;8.6]	0.784
Hematocrit (%)	0.4 [0.3;0.4]	0.4 [0.4;0.4]	0.747
Sodium (mmol/L)	138 [134;140]	137 [133;139]	0.022
Potassium (mmol L^−1^)	4.4 [4.0;4.7]	4.2 [3.9;4.6]	0.007
Creatinine (mmol/L)	118 [94;167]	90 [75;120]	< 0.001
eGFR (mL/min/1.73 m^2^)—CKD EPI	52 [33;72]	55 [39;74]	0.236
NT-proBNP (ng/L)	5083 [2726;9758]	4354 [2464;8356]	0.148
Treatment			
ACEi/ARB/ARNI	97 (56%)	72 (52%)	0.531
ACEi	56 (33%)	34 (25%)	0.161
ARB	27 (16%)	29 (21%)	0.289
ARNI	14 (8%)	9 (7%)	0.747
Beta-blockers	122 (71%)	93 (67%)	0.584
MRA	55 (32%)	52 (38%)	0.353
SGLT2i	10 (6%)	10 (7%)	0.781
Home loop diuretic	100 (58%)	77 (56%)	0.765
Home loop diuretic dose (mg of bumetanide equivalents)	2 [1;4]	2 [1;4]	0.208
ICD	45 (26%)	23 (17%)	0.061
CRT	20 (12%)	9 (7%)	0.181
Congestion			
Rales			0.247
Absent	44 (26%)	41 (30%)	
< 1/3 lungs	81 (49%)	52 (39%)	
1/3 to 2/3 lungs	30 (18%)	34 (25%)	
> 2/3 lungs	12 (7%)	8 (6%)	
Edema			0.208
Absent/trace	51 (31%)	46 (34%)	
Slight	27 (16%)	17 (13%)	
Moderate	45 (27%)	26 (19%)	
Marked	42 (25%)	45 (34%)	
JVP			0.905
Elevated	47 (37%)	43 (39%)	
Normal	24 (19%)	19 (17%)	
Unknown	57 (45%)	47 (43%)	
Orthopnoea	98 (67%)	76 (74%)	0.287
Nycturia	30 (34%)	12 (19%)	0.064

*ACEi* angiotensin-converting enzyme inhibitor, *ARB* angiotensin receptor blocker, *ARNI* angiotensin receptor-neprilysin inhibitor, *BMI* body mass index, *CKD* chronic kidney disease, *CRT* cardiac resynchronization therapy, *eGFR* estimated glomerular filtration rate, *HF* heart failure, *HFrEF* heart failure with reserved ejection fraction, *HFpEF* heart failure with preserved ejection fraction, *ICD* implantable cardioverter-defibrillator, *LVEF* left ventricular ejection fraction, *MRA* mineralocorticoid receptor antagonist, *NT-proBNP* N-terminal pro-B-type natriuretic peptide, *NYHA* New York Heart Association, *SGLT2i* sodium-glucose cotransporter 2 inhibitor

**Table 2:** Primary and secondary outcomes of men and women in PUSH-AHF

	Men			Women			
	All ***N*** = 172	Natriuresis-guided ***N*** = 89	SOC ***N*** = 83	All ***N*** = 138	Natriuresis-guided ***N*** = 61	SOC ***N*** = 77	Estimate (95% CI) ***p***-value for sex Interaction
Dual primary endpoint							
24-h natriuresis (mmol)							
* N*	151	78	73	127	53	74	68 (− 22 to 158) Unadjusted: 0.140 48 (− 39 to 135) Adjusted: 0.276
Mean (SD)	400 (189)	443 (176)	354 (192)	345 (195)	357 (170)	336 (211)	
Difference (95% CI)		89 (30–148) *p* = 0.004			21 (− 45 to 88) *p* = 0.533		
180-day HF rehospitalization or all-cause mortality							
* N*	171	89	82	138	61	77	
Event	63 (37%)	28 (32%)	35 (43%)	33 (24%)	18 (30%)	15 (20%)	
Unadjusted HR (95% CI)		0.63 (0.38–1.04) *p* = 0.069			1.59 (0.80–3.16) *p* = 0.182		2.53 (1.09–5.91) 0.031
Adjusted HR (95% CI)		0.51 (0.30–0.86) *p* = 0.011			1.40 (0.67–2.91) *p* = 0.368		2.75 (1.13–6.72) 0.026
Secondary endpoints							
24-h diuresis (mL)							
* N*	151	78	73	127	53	74	561 (− 187 to 1310) Unadjusted: 0.141 321 (− 424 to 1067) Adjusted: 0.396
Median [IQR]	3790 [3170;4905]	4100 [3561;5196]	3400 [2850;4520]	3300 [2500;4470]	3480 [2850;4460]	3105 [2400;4450]	
Difference (95% CI)		700 (385–1220) *p* = 0.001			375 (− 330 to 924) *p* = 0.237		
48-h diuresis (mL)							
* N*	138	71	67	113	47	66	925 (− 142 to 1193) Unadjusted: 0.089 803 (− 285 to 1892) Adjusted: 0.147
Median [IQR]	6615 [5300;7862]	7160 [5780;8382]	6050 [4750;7388]	5700 [4500;7200]	5700 [4838;7075]	5830 [4405;7540]	
Difference (95% CI)		1110 (490–1937) *p* = 0.003			− 130 (− 930 to 990) *p* = 0.870		
48-h natriuresis (mmol)							
* N*	138	71	67	113	47	66	110 (− 26 to 246) Unadjusted: 0.113 111 (− 25 to 247) Adjusted: 0.108
Mean (SD)	639 (251)	698 (236)	577 (252)	578 (297)	585 (254)	574 (326)	
Difference (95% CI)		121 (40–203) *p* = 0.004			11 (− 96 to 119) *p* = 0.835		
Length of hospital stay (days)							
* N*	171	89	82	138	61	77	8 (3–13) Unadjusted: 0.003 7 (2–12) Adjusted: 0.005
Median [IQR]	7 [5;10]	6 [5;9]	8 [5;12]	7 [5;9]	6 [5;10]	7 [5;9]	
* p*-value		0.019			0.866		
In-hospital death							
* N*	172	89	83	138	61	77	Unadjusted: 0.992 Adjusted: 0.990
Event	10 (6%)	0	10 (12%)	4 (3%)	2 (3%)	2 (3%)	
* p*-value		< 0.001			1.000		
HF rehospitalization (***n*** (%))							
* N*	171	89	82	138	61	77	
Event	32 (19%)	15 (17%)	17 (21%)	19 (14%)	10 (16%)	9 (12%)	
Unadjusted HR (95% CI)		0.68 (0.34–1.37) *p* = 0.283			1.48 (0.60–3.64) *p* = 0.396		046 (0.15–1.44) 0.184
Adjusted HR (95% CI)		0.58 (0.28–1.19) *p* = 0.138			1.54 (0.6–3.97) *p* = 0.371		039 (0.12–1.26) 0.161
Total number of HF rehospitalizations per patient							
* N*	32	15	17	19	10	9	0.6 (− 0.1 to 1.3) Unadjusted: 0.082 0.8 (0.1–1.5) Adjusted: 0.021
Median [IQR]	1.00 [1.00;1.25]	1.00 [1.00;1.00]	1.00 [1.00;2.00]	1.00 [1.00;1.50]	1.00 [1.00;2.00]	1.00 [1.00;1.00]	
* p*-value		0.454			0.152		
180-day all-cause mortality							
* N*	171	89	82	138	61	77	
Event	43 (25%)	20 (23%)	23 (28%)	19 (14%)	9 (15%)	10 (13%)	
Unadjusted HR (95% CI)		0.72 (0.40–1.31) *p* = 0.283			1.14 (0.46–2.81) *p* = 0.776		0.63 (0.21–1.85) 0.396
Adjusted HR (95% CI)		0.61 (0.33–1.14) *p* = 0.122			0.91 (0.33–2.48) *p* = 0.85		0.7 (0.22–2.26) 0.556
Percentage change in NT-proBNP (%)							
At 48 h	− 20 [− 44;19]	− 23 [− 50;12]	− 12 [− 38;24]	− 19 [− 43;13]	− 21 [− 42;12]	− 19 [− 45;15]	20 (− 42 to 83) Unadjusted: 0.524 15 (− 49 to 78) Adjusted: 0.650
* p*-value		0.365			0.941		
At 72 h	− 34 [− 60;7]	− 37 [− 65;2]	− 30 [− 52;11]	− 30 [− 59; − 6]	− 29 [− 55; − 5]	− 33 [− 60; − 7]	5 (–56 to 66) Unadjusted: 0.884 4 (–57 to 66) Adjusted: 0.886
* p*-value		0.325			0.549		
Natriuresis, diuretic dose and natriuretic response							
Poor natriuresis (24 h natriuresis < 340.5 mmol)							
* n* (%)	64 (42%)	24 (31%)	40 (55%)	75 (59%)	27 (51%)	48 (65%)	OR 0.65 (0.24–1.74) Unadjusted: 0.392 OR 0.73 (0.24–2.21) Adjusted: 0.574
* p*-value		0.005			0.164		
24-h loop diuretic dose (mg of bumetanide equivalents)							
* n*	171	89	82	137	61	76	2 (− 1 to 4) Unadjusted: 0.240 − 1.31 (− 3.6 to 1) Adjusted: 0.263
Median (IQR)	8 [6;15]	12 [7;19]	6 [5;11.5]	8 [5;15]	13 [7.50;19]	6 [3;12]	
* p*-value		< 0.001			< 0.001		
24-h natriuretic response (mmol/mg)							
Median (IQR)	39 [21;83]	38 [19;76]	45 [23;85]	36 [17;73]	23 [16;45]	52 [25;99]	− 35 (− 59 to − 10) Unadjusted: 0.007 − 32 (− 56 to − 8) Adjusted: 0.009
* p*-value		0.377			0.001		

Reference for estimate (95% CI) is Male sex and Standard of Care for the interaction analysis. Adjusted *p*-value: adjusted for baseline differences between men and women: heart rate, heart failure etiology, myocardial infarction, sodium, potassium, creatinine. *OR* odds ratio

### Effects of natriuresis-guided therapy in men and women

#### Dual primary endpoint

**Fig. 1:**
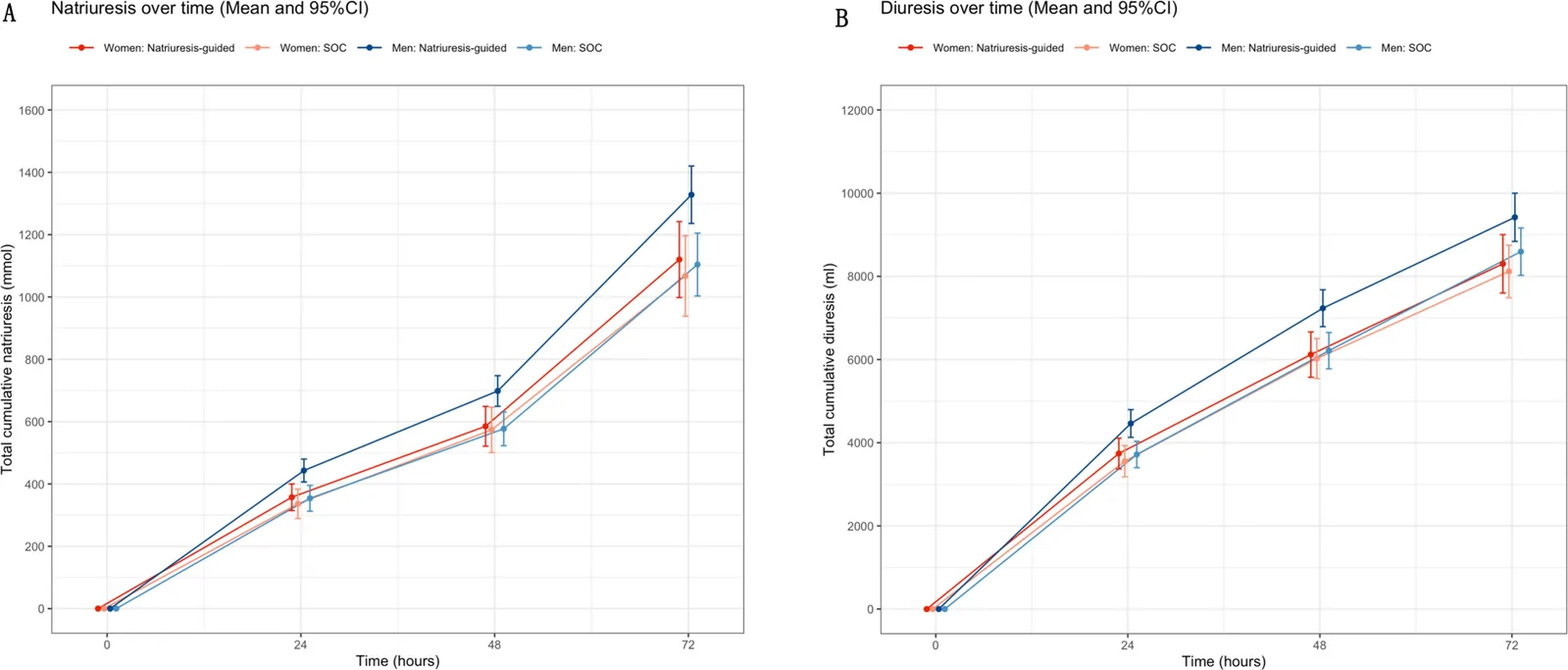
Total cumulative natriuresis (**A**) and diuresis (**B**) over time. **A** Total cumulative natriuresis (mmol) within the first 72 h, shown in 4 groups defined by treatment allocation and sex. **B** Total cumulative diuresis (mL) within the first 72 h, shown in 4 groups defined by treatment allocation and sex. Abbreviations: CI, confidence interval; SOC, standard of care

**Fig. 2:**
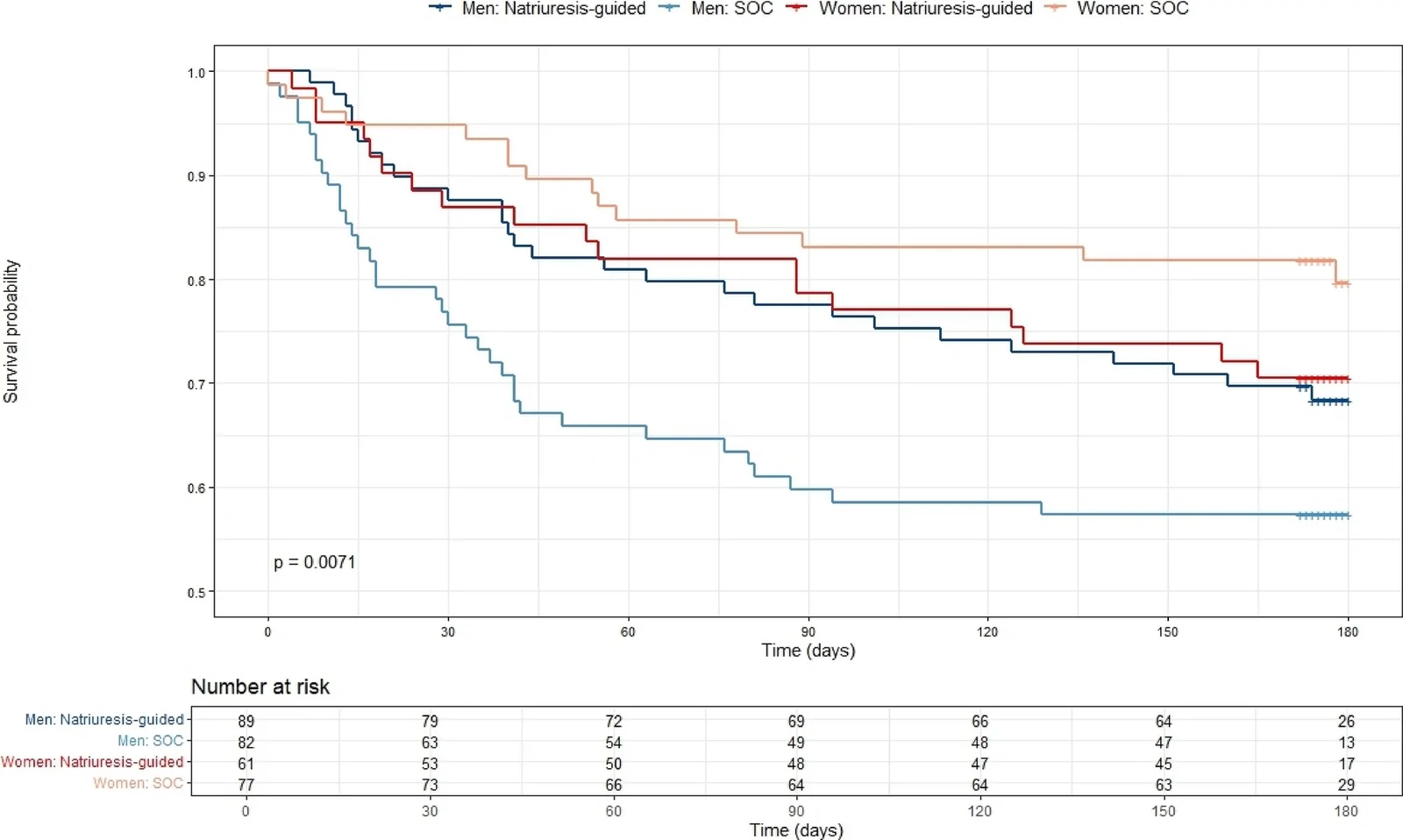
Kaplan–Meier curves: 180 days all-cause mortality and heart failure rehospitalization in men and women. Kaplan–Meier curve for the composite outcome of HF rehospitalization and all-cause mortality after 180 days, shown in 4 groups defined by treatment allocation and sex. Adjusted HR in men, 0.51 (0.30–0.86), *p* = 0.01, natriuresis-guided therapy is used as reference. Adjusted HR in women, 1.40 (0.67–2.91), *p* = 0.368, natriuresis-guided therapy is used as reference. *p*-interaction (sex × treatment allocation) = 0.026. Abbreviations: SOC, standard of care

For the first part of the dual primary endpoint, mean total 24-h natriuresis was higher in natriuresis-guided diuretic therapy compared to SOC in men (443 vs. 354 mmol, mean difference 89 mmol (95% CI), 30–148, *p* = 0.004), whereas in women, mean total 24-h natriuresis was comparable (357 vs. 336 mmol, mean difference 21 mmol (95% CI), − 45 to 88, *p* = 0.533). Treatment effect was however consistent across men and women, with no significant treatment interaction (adjusted *p*-interaction = 0.276) (Table 2, Fig. 1). For the second part of the dual primary endpoint, natriuresis-guided diuretic therapy significantly reduced the risk of 180-day HF rehospitalization or all-cause mortality in men (adjusted HR (95% CI), 0.51 (0.30–0.86)), but not in women (adjusted HR (95% CI), 1.40 (0.67–2.91)), with significant interaction between effect of treatment and sex (adjusted *p*-interaction = 0.026) (Table 2). Figure 2 presents the Kaplan–Meier curves for the second dual primary endpoint in men and women, illustrating that men in the SOC treatment arm had worse composite outcomes of heart failure rehospitalization or all-cause death at 180 days.

#### Secondary endpoint

The trajectories of total natriuresis and diuresis from baseline to 72 h in men and women are shown in Fig. 1. Men in the natriuresis-guided treatment arm demonstrated the highest total natriuresis and diuresis over time compared with other groups. The effect of natriuresis-guided diuretic therapy was consistent in men and women for total 24-h and 48-h diuresis, 48-h natriuresis, HF rehospitalization, in-hospital death, and all-cause mortality (all *p*-interaction > 0.1) (Table 2). In-hospital death was much higher in men with SOC compared to men with natriuresis-guided diuretic therapy (12% vs. 0%, *p* < 0.001). Additionally, the median length of hospital stay was significantly shorter in men with natriuresis-guided diuretic therapy compared with men with SOC (6 vs. 8 days, *p* = 0.019), whereas in women, it was similar between both arms (6 vs. 7 days, *p* = 0.886, respectively). Sex significantly modified the association between the treatment effect and length of hospital stay (*p*-interaction = 0.005) (Table 2). The proportions of patients who experienced adverse events were similar between treatment groups in both men and women (Supplemental Table 3).

**Table 3:** Changes in renal function, the association between worsening renal function at day 3 and 180-day primary outcome in men and women

	Men			Women			
	All	Natriuresis-guided	SOC	All	Natriuresis-guided	SOC	
eGFR (mL/min/1.73 m^2^)							
Baseline	52 [33;72]	54 [35;72]	50 [33;70]	55 [39;74]	55 [34;72]	55 [42;75]	
* p*-value		0.688		0.236	0.635		
24 h	48 [33;63]	48 [34;62]	47 [32;64]	50 [37;68]	47 [32;67]	53 [38;70]	
* p*-value		0.967		0.255	0.272		
48 h	42 [29;55]	42 [29;53]	44 [30;58]	48 [34;65]	42 [29;58]	52 [37;70]	
* p*-value		0.542		0.027	0.032		
72 h	40 [28;57]	39 [28;54]	48 [27;60]	50 [33;62]	40 [26;56]	52 [35;68]	
* p*-value		0.434		0.071	0.010		
Discharge	40 [29;55]	39 [30;53]	42 [27;56]	48 [33;62]	43 [29;58]	51 [37;64]	
* p*-value		0.987		0.016	0.056		
Relative change in eGFR (%)							
24 h	− 5 [− 15;2]	− 8 [− 17;1]	− 3 [− 12;3]	− 5 [− 17;3]	− 7 [− 17;0]	− 4 [− 16;8]	
* p*-value		0.182		0.975	0.182		
48 h	− 13 [− 26;0]	− 15 [− 28; − 3]	− 12 [− 20;2]	− 12 [− 25;4]	− 18 [− 25; − 2]	− 9 [− 22;9]	
* p*-value		0.070		0.344	0.040		
72 h	− 16 [− 30;0]	− 18 [− 30; − 5]	− 14 [− 30;1]	− 14 [− 29;2]	− 19 [− 33; − 4]	− 10 [− 25;5]	
* p*-value		0.279		0.498	0.030		
Discharge	− 19 [− 31; − 2]	− 20 [− 30;0]	− 18 [− 33; − 4]	− 13 [− 27;1]	− 20 [− 30;1]	− 10 [− 24;1]	
* p*-value		0.998		0.076	0.108		
Increase in serum creatinine ≥ 26.5 μmol/L (0.3 mg/dL) between baseline and 72 h							***p*** for interaction
* N* (%)	61 (39%)	35 (42%)	26 (36%)	36 (30%)	22 (42%)	14 (21%)	
* p*-value		0.501			0.022		
Unadjusted HR (95% CI)		0.74 (0.42–1.30) *p* = 0.298			1.38 (0.61–3.11) *p* = 0.440		0.097
Adjusted HR (95% CI)		0.8 (0.45–1.42) *p* = 0.441			1.29 (0.48–3.50) *p* = 0.614		0.132
Increase in serum creatinine ≥ 26.5 µmol/L (0.3 mg/dL) and increase > 25% between baseline and 72 h							
* N* (%)	50 (32%)	30 (37%)	20 (27%)	34 (28%)	20 (38%)	14 (21%)	
p-value		0.294			0.060		
Unadjusted HR (95% CI)		0.79 (0.43–1.43) *p* = 0.436			1.25 (0.55–2.86) *p* = 0.593		0.196
Adjusted HR (95% CI)		0.81 (0.44–1.47) *p* = 0.480			1.17 (0.41–3.29) *p* = 0.770		0.199
Decrease in eGFR > 25% between baseline and 72 h							
* N* (%)	50 (32%)	28 (34%)	22 (30%)	40 (33%)	22 (42%)	18 (26%)	
* p*-value		0.714			0.121		
Unadjusted HR (95% CI)		0.79 (0.43–1.42) *p* = 0.425			1.42 (0.64–3.13) *p* = 0.389		0.134
Adjusted HR (95% CI)		0.82 (0.45–1.49) *p* = 0.507			1.29 (0.47–3.51) *p* = 0.617		0.200

Adjusted *p*-value: adjusted for baseline differences between men and women: heart rate, heart failure etiology, myocardial infarction, sodium, potassium, creatinine

### Natriuretic response in men and women

Overall, women were more likely to have a poor 24-h natriuresis (below the median) compared to men (75 (59%) vs. 64 (42%)), *p* = 0.008; adjusted *p*-interaction = 0.574, Table 2). However, the 24-h loop diuretic dose (in mg of bumetanide equivalents) was similar between men and women: 8 mg [6–15] vs. 8 [5–15], respectively (*p* = 0.665). Over 36 h, loop diuretic doses were higher in the natriuresis-guided arm compared to SOC arm in both men (*p* < 0.001) and women (*p* < 0.001), with no significant sex interaction (*p*-interaction = 0.504 in linear mixed modelling, Supplemental Fig. 1). Compared to SOC, the median 24-h natriuretic response was lower in women receiving natriuresis-guided therapy (23 vs. 52 mmol urinary sodium/mg bumetanide, *p* = 0.001), whereas in men, there was no significant difference between treatment arms (adjusted *p*-interaction = 0.009, Table 2).

### Changes in eGFR and WRF in men and women

**Fig. 3:**
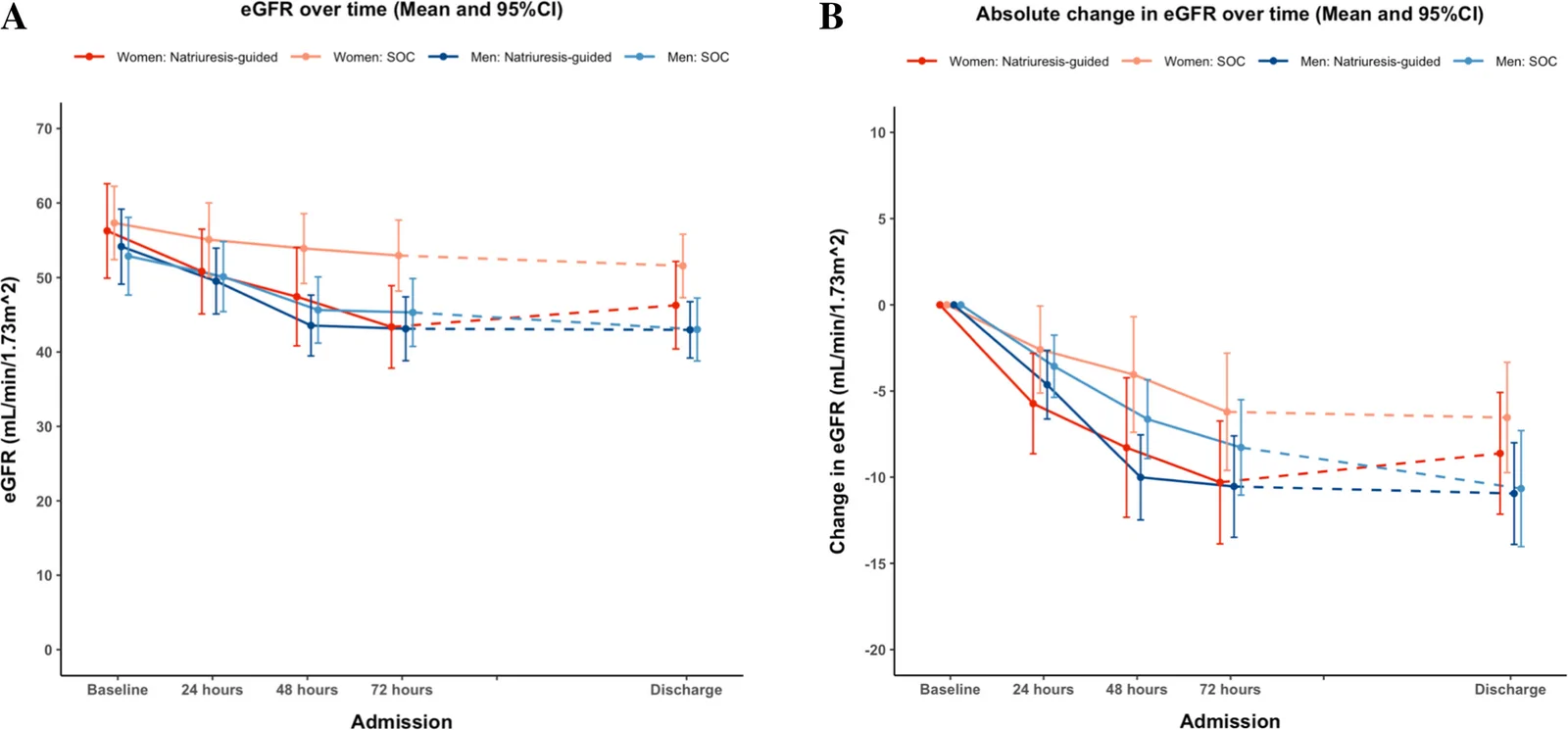
eGFR at each time point (**A**) and delta eGFR (**B**) over the admission in men and women across treatment arms. Left panel: eGFR over time, shown in 4 groups defined by treatment allocation and sex. Right panel: Absolute change in eGFR over time, shown in the same groups. Mean values are shown with dots, and the bars represent 95% CI. *p*-value were calculated in linear mixed-effect models. Men (natriuresis-guided treatment vs. SOC): ANOVA main *p*-value for difference between the models (*p* = 0.467). Women (natriuresis-guided treatment vs. SOC): ANOVA main *p*-value for difference between the models (*p* = 0.017). *p*-interaction (sex × treatment allocation) = 0.005. Abbreviations: CI, confidence interval; eGFR, estimated glomerular filtration rate; SOC, standard of care

**Fig. 4:**
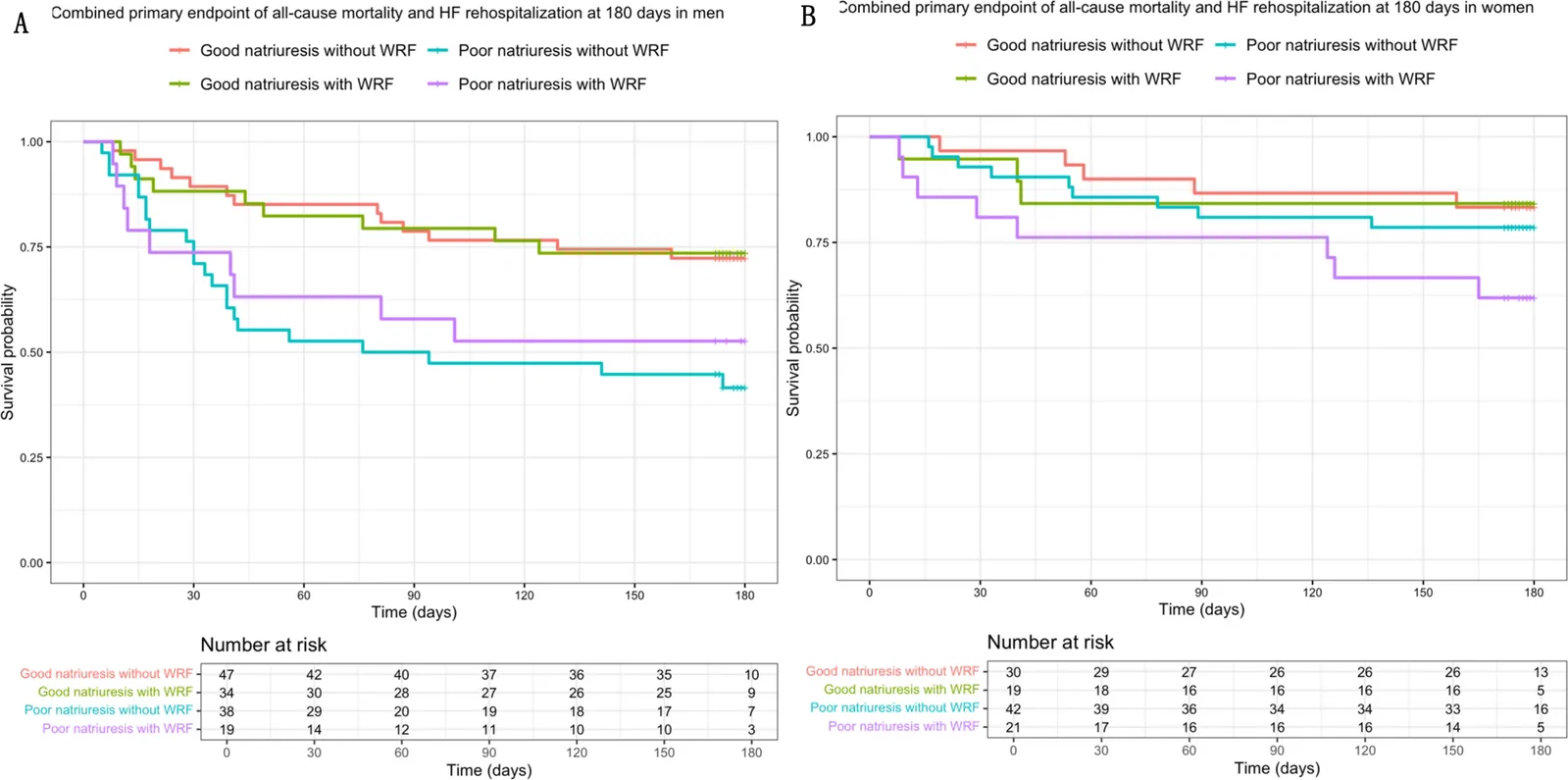
Kaplan–Meier curve for good/poor natriuresis with and without WRF and 180-day primary endpoints in men (left, **A**) and women (right, **B**). Kaplan–Meier curves are shown for 4 groups, defined by good or poor natriuresis (below or above the median) and the occurrence of worsening renal function. **A**: curves shown for men, group differences *p* = 0.005. **B**: curves shown for women, group differences, *p* = 0.220. Abbreviations: WRF, worsening renal function

Table 3 shows eGFR and relative changes in eGFR from baseline to discharge in men and women. In men, natriuresis-guided diuretic therapy did not result in significant changes in eGFR at any time point during hospitalization compared to SOC (*p* = 0.467, Fig. 3 and Table 3). However, women receiving natriuresis-guided therapy showed significant changes in eGFR over time compared with SOC (*p* = 0.017), with significant *p* for interaction 0.005 (Fig. 3). Women receiving natriuresis-guided therapy more often experienced WRF (increase in serum creatinine ≥ 26.5 μmol/L (0.3 mg/dL) between baseline and 72 h) compared to women with SOC (42% vs. 14%, respectively, *p* = 0.022). However, when WRF was additionally required to include a > 25% in serum creatinine, this difference was not significant (*p* = 0.060). WRF was not associated with an increased risk of the composite outcome of all-cause mortality or HF rehospitalization at 180 days in either men or women, irrespective of treatment allocation (Table 3). Moreover, there was no evidence of a sex interaction between WRF and worse clinical outcomes (Table 3). Kaplan–Meier curves are shown for good or poor natriuresis with and without WRF in men (Fig. 4A) and women (Fig. 4B). Poor 24-h natriuresis without WRF was associated with worse 180-day outcomes in men (adjusted HR (95% CI), 3.31 (1.61–6.79), *p* = 0.001), but not in women; however, there was no significant interaction (Supplemental Table 4).

## Discussion

The primary outcomes of the PUSH-AHF trial showed a significant sex-specific interaction for the overall neutral endpoint of 180-day HF rehospitalization or all-cause mortality, with a potential benefit of natriuresis-guided diuretic therapy observed in men but not in women. In this sex-specific subgroup analysis, women were more likely to exhibit a lower natriuretic response during hospitalization compared to men. After adjustment for baseline differences, natriuresis-guided diuretic therapy was associated with a reduced risk of 180-day HF rehospitalization and/or all-cause mortality in men, whereas no such association was seen in women. These findings, however, should be interpreted with caution given the post hoc nature of this analysis. Although WRF was more frequently observed in women receiving natriuresis-guided diuretic therapy compared with SOC, it was not associated with worse clinical outcomes.

### Baseline characteristics between men and women with acute heart failure

Consistent with findings from previous large acute HF registries [3, 7, 16] and trials [8, 11, 17], PUSH-AHF showed classic sex-related differences in presentation, characterized by a more frequent ischemic etiology of HF in men. In contrast with previous studies, we found that men and women in PUSH-AHF were of similar age, with similar body mass index and systolic blood pressure. Baseline diuretic use was comparable between men and women in PUSH-AHF, which is in line with data from ADHERE [7], ASCEND-HF [8], EVEREST [17], REPORT-HF [16], and EHFS II [18]. Furthermore, we observed that there were no differences in the signs and symptoms of HF between men and women, which is also consistent with previously published data [7, 8].

### Efficacy of natriuresis-guided therapy in men and women

#### Natriuresis and diuresis

Natriuresis is increasingly used as a marker of diuretic response with lower values indicating a poorer response [19]. In the present study, no significant treatment interaction was observed for the efficacy of natriuresis-guided diuretic therapy between men and women. When analyzed separately, natriuresis-guided therapy was associated with enhanced natriuresis and diuresis in men, whereas these differences were less pronounced in women. However, given the absence of a statistically significant treatment interaction, these findings do not provide solid evidence of treatment heterogeneity by sex. Overall, men were more likely to experience good natriuresis during hospitalization, and those receiving natriuresis-guided diuretic therapy achieved the highest cumulative natriuresis and diuresis from baseline to 72 h. This finding aligns with a previous prospective cohort study of 175 acute HF patients, which reported that female sex was associated with lower 6-h urinary sodium concentration (standardized beta = − 0.169, *p* = 0.013) following loop diuretic therapy [20]. Similarly, in the ADVOR trial, female sex was an independent predictor of poor total 48-h natriuresis (unstandardized beta = − 47, *p* = 0.034) [21].

#### Loop diuretic dose and natriuretic response

In the PUSH-AHF trial, there were no sex differences observed in median loop diuretic dose (bumetanide equivalents) between baseline and 36 h across both treatment arms. This data is consistent with the results from the ADHERE study [7] but contrasts with other studies reporting lower dosing in women [8, 10, 11]. A plausible explanation may relate to the standardized dosing protocol of loop diuretics in both randomization arms. Although sex-specific pharmacokinetic parameters for bumetanide are lacking, differences have been reported for torsemide, another loop diuretic: women demonstrated a 1.5-fold higher 24-h area under the concentration–time curve, a 1.3-fold higher peak plasma concentration, and one-third lower oral clearance than men [22]. These differences suggest prolonged drug exposure in women, which could theoretically enhance diuretic efficacy. However, we observed a significant interaction between sex and 24-h natriuretic response: women receiving natriuresis-guided therapy had a poorer 24-h natriuretic response compared with SOC, whereas no difference was seen between treatment groups in men. As protocol-mandated dose escalation occurred in the natriuresis-guided arm when natriuresis targets were not achieved, this metric reflects sodium excretion efficiency under the applied treatment strategy rather than a pure measure of intrinsic diuretic responsiveness. Therefore, differences in natriuretic response between treatment groups may partly reflect treatment-related dose adjustments. Nevertheless, our findings are in line with observations from the ASCEND-HF trial; female sex was independently associated with poor diuretic response, defined as weight change from admission to 48 h per 40 mg furosemide or equivalent [23]. Moreover, in healthy subjects, men had a higher peak sodium excretion rate than women after adjusted for mg/kg of intravenous furosemide dose [24]. Even though conclusive evidence is currently lacking, these findings suggest that men exhibit a better diuretic or natriuretic response than women when receiving similar doses of diuretics and warrant further investigation. The absence of an improved natriuretic response in women despite protocol-driven dose escalation may indicate sex-related differences in the mechanisms underlying diuretic resistance. Consequently, the observed sex-specific patterns do not support withholding natriuresis-guided diuretic therapy from women. Rather, they highlight the need for prospective investigation of whether the optimal marker, threshold, dosing strategy, or renal tolerance of decongestion may differ according to sex and HF phenotype. Given the exploratory nature of these analyses, this hypothesis requires confirmation in future studies.

#### 180-day clinical outcomes

Men in the natriuresis-guided diuretic therapy arm had a significant reduction in the risk of 180-day HF rehospitalization or all-cause mortality compared with those receiving SOC. In contrast, this treatment benefit was not observed in women. This absence of effect in women may partly be reflected in their overall lower risk, regardless of treatment allocation. Additionally, it may be related to the fact that men in the natriuresis-guided diuretic therapy arm achieved more increased natriuresis, diuresis, and effective decongestion during hospitalization even though evidence for a significant treatment interaction was not consistent across these endpoints. A meta-analysis of 19 studies revealed that higher urinary sodium concentration was associated with increased urinary output, greater weight loss, shorter hospital length of stay, and lower mortality [25]. Consistent with these observations, men receiving natriuresis-guided diuretic therapy in our study had a shorter hospital stay and no in-hospital deaths compared to men in the SOC group. However, given the limited sample size and low numbers of events in these analyses, these findings should be interpreted with caution. The apparent benefit observed in men may partly reflect poorer outcomes in the SOC group, and the possibility of chance findings, residual confounding, or limited statistical power cannot be excluded. Therefore, the observed sex-specific differences should be considered exploratory and hypothesis-generating and require confirmation in adequately powered prospective studies. The potential mechanism underlying the differential treatment response between men and women cannot be determined from the present data; however, several explanations and hypotheses warrant consideration. This treatment heterogeneity may partly be explained by differences in HF phenotypes; men with HF more predominantly present with HFrEF, whereas women more commonly present with HFpEF. In a subgroup analysis of the ROSE-AHF (Renal Optimization Strategies Evaluation in Acute Heart Failure) trial, differential effects on decongestion were observed between HFrEF and HFpEF patients, with HFrEF patients demonstrating increased total 72-h urine volume, whereas no such improvement was observed in HFpEF patients [26]. In PUSH-AHF, we were unable to confirm this observation because baseline ejection fraction was missing in 25% of patients. Consequently, adjustment for phenotype was not feasible. This limitation is particularly relevant because women in our cohort had a numerically higher prevalence of HFpEF. Therefore, it cannot be excluded that the apparent differences between men and women were at least partly driven by differences in HF phenotype. Among the available baseline data on signs and symptoms of congestion, we found no significant differences in congestion phenotypes between men and women. Both sexes presented with similar clinical signs, symptoms, and NT-proBNP levels.

Due to the limited sample size in the subgroup analysis, large-scale multi-center trials are needed to explore these observations and further clarify the sex-specific differences in outcomes. Finally, the overall risk of 180-day mortality and HF rehospitalization was markedly lower in women than men, which deserves further exploration in larger scale trials.

### Worsening renal function and safety of natriuresis-guided therapy in men and women

Several studies have reported that women more frequently experience WRF than men during hospital stay [10, 27, 28]. For instance, in the PROTECT trial, the proportion of women with WRF was approximately 10% higher compared with men [28]. Similarly, in the CLOROTIC trial, a higher incidence of WRF was observed in women compared to men; however, this was not associated with an increased risk of rehospitalization or mortality at 90 days [27]. In contrast, in the RELAX-AHF-2 trial, early WRF was more frequent in women and was associated with worse 180-day outcomes [10]. In our study, women allocated to natriuresis-guided diuretic therapy were more likely to develop WRF compared with women treated with SOC. The greatest decline in eGFR in women receiving natriuresis-guided therapy occurred within the first 24 h of treatment, followed by a more gradual decline through 72 h, a trajectory broadly consistent with that observed in men. In contrast, women receiving SOC showed minimal changes in eGFR over the same period. However, WRF itself was not associated with worse clinical outcomes in this analysis. Despite concerns that women may experience more adverse drug reactions and are more likely to discontinue treatment [29], previous evidence also indicates a 13% higher relative risk of hospital admissions due to loop diuretic–related adverse events in women compared with men, even after adjusting for prescription frequency [30]. However, the safety data from PUSH-AHF were reassuring. Taken together, our data support that natriuresis-guided diuretic therapy is a safe and well-tolerated treatment in patients with acute HF, irrespective of sex. Given the small number of events, however, the observed changes in renal function, particularly among women, warrant confirmation in larger studies.

### Limitations

These analyses are subject to several limitations; therefore, the results should be interpreted with caution and considered hypothesis-generating. First, the assessment of clinical endpoints by sex was conducted post hoc, even though sex was a predefined subgroup. PUSH-AHF was not specifically designed to ensure sufficient statistical power for sex subgroup analyses; therefore, the present study should be regarded as exploratory and hypothesis-generating. While the remarkable observed difference may be attributable to chance, it nonetheless does warrant further investigation. Second, although PUSH-AHF was an open-label pragmatic trial designed to closely mimic real-world clinical practice, some inherent limitations of this design include a higher rate of missing data (e.g., around 25% of patients missing LVEF data), protocol deviations, and the lack of a systematic assessment of congestion status. Third, dietary sodium intake is an important determinant of urinary sodium excretion; however, this was not accounted for in the study design, given its pragmatic nature. This may have introduced unmeasured variability and potential bias. Finally, the PUSH-AHF trial was a single-center trial in the Netherlands, which may limit the diversity of the study population. These limitations could impact the generalizability of our findings.

## Conclusions

Potential sex-related differences in the response to natriuresis-guided diuretic therapy were observed. Natriuresis-guided diuretic therapy appears less effective in improving natriuretic response and resulted in more frequent WRF in women. In contrast, men seem to derive greater benefit, demonstrating improved natriuresis and significant reduction 180-day all-cause mortality or HF rehospitalization risk. These findings warrant further investigation and confirmation to clarify the sex-specific differences in response to diuretic therapy in patients with acute HF.

## Supplementary Information

Below is the link to the electronic supplementary material.

ESM 1 (DOCX 238 KB)

## Data Availability

Anonymized participant data can be made available upon request directed to the corresponding author. Proposals will be reviewed on the basis of scientific merit, ethical review, available resources, and regulatory requirements. After approval of a proposal, anonymized data will be made available for reuse. A steering committee will have the right to review and comment on any draft papers based on these data before publication.

## Abbreviations

ACEi: Angiotensin-converting enzyme inhibitor
ADHERE: Acute Decompensated Heart Failure National Registry
ARB: Angiotensin receptor blocker
ASCEND-HF: Acute Study of Clinical Effectiveness of Nesiritide in Decompensated Heart Failure
CLOROTIC: Safety and Efficacy of the Combination of Loop with Thiazide-type Diuretics in Patients with Decompensated Heart Failure
eGFR: Estimated glomerular filtration rate
ESCAPE: Evaluation Study of Congestive Heart Failure and Pulmonary Artery Catheterization Effectiveness
HF: Heart failure
HFpEF: Heart failure with preserved ejection fraction
HFrEF: Heart failure with reduced ejection fraction
LVEF: Left ventricular ejection fraction
PROTECT: Placebo-controlled Randomized study of the selective A1 adenosine receptor antagonist rolofylline for patients hospitalized with acute decompensated heart failure and volume Overload to assess Treatment Effect on Congestion and renal function
PUSH-AHF: Pragmatic Urinary Sodium-based treatment algoritHm in Acute Heart Failure
RCT: Randomized controlled trial
RELAX-AHF: Serelaxin, recombinant human relaxin-2, for treatment of acute heart failure
RELAX-AHF-2: Efficacy, Safety and Tolerability of Serelaxin When Added to Standard Therapy in AHF
ROSE-AHF: Renal Optimization Strategies Evaluation in Acute Heart Failure
SD: Standard deviation
SOC: Standard of care
UMCG: University Medical Center Groningen
