# Supplementary material for: Effects of natriuresis-guided diuretic therapy in men and women with acute heart failure: insights from PUSH-AHF
Source: Clin Res Cardiol. 2026 Sep 28;115(11):2062–76. doi: 10.1007/s00392-026-03030-1 (PMC13658324; doi:10.1007/s00392-026-03030-1)
Supplement: Supplementary file 1 [file 392_2026_3030_MOESM1_ESM.docx]

**Supplemental Material**

**Supplemental Table 1. Baseline characteristics for men and women, stratified by NGT and SOC**

**Supplemental Table 2. Baseline characteristics for treatment allocation, stratified by sex**

**Supplemental Table 3. Cox regression analysis for good/poor natriuresis response with and without WRF to the 180-day primary endpoints**

**Supplemental Table 4. Safety endpoints according to treatment group and sex**

**Supplemental Figure 1. Total loop diuretic doses over time (Mean and 95% CI)**

**Supplemental Figure 2**. **Total cumulative natriuresis over eGFR at baseline in different groups of men and women**

**Supplemental Table 1. Baseline characteristics for men and women, stratified by NGT and SOC**

|  | **Men** | | | **Women** | | |
| --- | --- | --- | --- | --- | --- | --- |
|  | **Natriuresis guided therapy N=89** | **Standard of care N=83** | **p.overall** | **Natriuresis guided therapy N=61** | **Standard of care N=77** | **p.overall** |
| **Demographics** |  |  |  |  |  |  |
| Age (in years) at time of screening | 73 [66;80] | 72 [64;80] | 0.918 | 75 [66;84] | 76 [65;83] | 0.711 |
| Race, Caucasian | 87 (98%) | 79 (95%) | 0.709 | 55 (93%) | 76 (100%) | 0.034 |
| **Physical examination** |  |  |  |  |  |  |
| BMI (kg/m2) | 29 (4) | 27 (6) | 0.293 | 27 (6) | 30 (8) | 0.076 |
| Systolic blood pressure (mmHg) | 130 (25) | 133 (28) | 0.366 | 133 (29) | 130 (25) | 0.524 |
| Diastolic blood pressure (mmHg) | 80 (16) | 82 (20) | 0.538 | 79 (18) | 80 (20) | 0.720 |
| Heart rate | 88 (25) | 90 (26) | 0.656 | 94 (29) | 103 (30) | 0.071 |
| NYHA class |  |  | 0.077 |  |  | 0.947 |
| Class II | 2 (2%) | 7 (8%) |  | 3 (5%) | 4 (5%) |  |
| Class III | 27 (30%) | 16 (19%) |  | 12 (20%) | 13 (17%) |  |
| Class IV | 60 (67%) | 60 (72%) |  | 46 (75%) | 60 (78%) |  |
| **HF characteristics** |  |  |  |  |  |  |
| LVEF (%) | 36 (14) | 36 (13) | 0.863 | 40 (14) | 40 (13) | 0.931 |
| HFpEF | 16 (24%) | 8 (13%) | 0.157 | 14 (29%) | 13 (26%) | 0.853 |
| Ischemic aetiology | 44 (49%) | 35 (42%) | 0.422 | 12 (20%) | 20 (26%) | 0.504 |
| New-onset HF | 36 (40%) | 36 (44%) | 0.763 | 30 (49%) | 33 (43%) | 0.570 |
| Time since HF diagnosis (months) | 10 [0;72] | 6 [0;94] | 0.662 | 0 [0;54] | 27 [0;74] | 0.344 |
| **Medical history** |  |  |  |  |  |  |
| Myocardial infraction | 36 (40%) | 32 (39%) | 0.922 | 12 (20%) | 20 (26%) | 0.504 |
| Hypertension | 59 (66%) | 51 (61%) | 0.615 | 34 (56%) | 51 (66%) | 0.279 |
| Atrial fibrillation | 48 (54%) | 48 (58%) | 0.718 | 36 (59%) | 42 (55%) | 0.724 |
| Diabetes | 31 (35%) | 37 (45%) | 0.250 | 21 (34%) | 29 (38%) | 0.830 |
| Depression | 4 (4%) | 7 (8%) | 0.457 | 1 (2%) | 5 (6%) | 0.228 |
| **Laboratory values** |  |  |  |  |  |  |
| Haemoglobin (mmol l^−1^) | 7.7 [6.9;8.7] | 7.7 [6.8;8.6] | 0.969 | 7.7 [6.8;8.5] | 7.9 [7.2;8.8] | 0.166 |
| Haematocrit (%) | 0.4 [0.3;0.4] | 0.4 [0.3;0.4] | 0.717 | 0.4 [0.3;0.4] | 0.4 [0.4;0.4] | 0.184 |
| Sodium (mmol/L) | 138 [134;140] | 137 [134;140] | 0.842 | 136 [133;139] | 137 [134;139] | 0.493 |
| Potassium (mmol l^−1^) | 4.5 [4.0;4.8] | 4.4 [4.0;4.7] | 0.285 | 4.2 [4.0;4.4] | 4.2 [3.9;4.6] | 0.911 |
| Creatinine (mmol/L) | 117 [93;163] | 122 [95;181] | 0.632 | 88 [74;128] | 94 [75;110] | 0.852 |
| eGFR (ml/min/1.73m2) - CKD EPI | 54 [35;72] | 50 [32;70] | 0.688 | 55 [34;72] | 55 [42;75] | 0.635 |
| NT-proBNP (ng/L) | 5816 [2515;9033] | 4995 [2788;11299] | 0.509 | 3860 [2652;7023] | 4931 [1975;8732] | 0.437 |
| **Treatment** |  |  |  |  |  |  |
| ACEi/ARB/ARNI | 50 (56%) | 47 (57%) | 1.000 | 32 (52%) | 40 (52%) | 1.000 |
| ACEi | 25 (28%) | 31 (37%) | 0.258 | 18 (30%) | 16 (21%) | 0.326 |
| ARB | 16 (18%) | 11 (13%) | 0.521 | 11 (18%) | 18 (23%) | 0.579 |
| ARNI | 9 (10%) | 5 (6%) | 0.483 | 3 (5%) | 6 (8%) | 0.731 |
| Beta-blockers | 62 (70%) | 60 (72%) | 0.833 | 38 (62%) | 55 (71%) | 0.340 |
| MRA | 32 (36%) | 23 (28%) | 0.320 | 21 (34%) | 31 (40%) | 0.599 |
| SGLT2i | 4 (4%) | 6 (7%) | 0.525 | 4 (7%) | 6 (8%) | 1.000 |
| Home loop diuretic | 49 (55%) | 51 (61%) | 0.488 | 35 (57%) | 42 (55%) | 0.873 |
| Home loop diuretic dose (mg of bumetanide equivalents) | 2 [1;4] | 2 [1;4] | 0.488 | 2 [1;4] | 2 [1;4] | 0.838 |
| ICD | 22 (25%) | 23 (28%) | 0.785 | 11 (18%) | 12 (16%) | 0.878 |
| CRT | 12 (13%) | 8 (10%) | 0.584 | 5 (8%) | 4 (5%) | 0.508 |
| **Congestion** |  |  |  |  |  |  |
| Rales |  |  | 0.922 |  |  | 0.428 |
| Absent | 23 (26%) | 21 (27%) |  | 18 (30%) | 23 (31%) |  |
| < 1/3 lungs | 41 (47%) | 40 (51%) |  | 26 (43%) | 26 (35%) |  |
| 1/3 to 2/3 lungs | 17 (19%) | 13 (16%) |  | 12 (20%) | 22 (30%) |  |
| >2/3 lungs | 7 (8%) | 5 (6%) |  | 5 (8%) | 3 (4%) |  |
| Edema |  |  | 0.315 |  |  | 0.383 |
| Absent/trace | 23 (27%) | 28 (35%) |  | 23 (38%) | 23 (32%) |  |
| Slight | 14 (17%) | 13 (16%) |  | 9 (15%) | 8 (11%) |  |
| Moderate | 28 (33%) | 17 (21%) |  | 8 (13%) | 18 (25%) |  |
| Marked | 19 (23%) | 23 (28%) |  | 21 (34%) | 24 (33%) |  |
| JVP |  |  | 0.250 |  |  | 0.936 |
| Elevated | 21 (34%) | 26 (39%) |  | 20 (40%) | 23 (39%) |  |
| Normal | 9 (15%) | 15 (23%) |  | 8 (16%) | 11 (19%) |  |
| Unknown | 32 (52%) | 25 (38%) |  | 22 (44%) | 25 (42%) |  |
| Orthopnoea | 49 (62.8%) | 49 (71%) | 0.381 | 36 (73%) | 40 (74%) | 1.000 |

ACEi, angiotensin-converting enzyme inhibitor; ARB, angiotensin receptor blocker; ARNI, angiotensin receptor–neprilysin inhibitor; BMI, body mass index; CKD, chronic kidney disease; CRT, cardiac resynchronization therapy; eGFR, estimated glomerular filtration rate; HF, heart failure; HFrEF, heart failure with reserved ejection fraction; HFpEF, heart failure with preserved ejection fraction; ICD, implantable cardioverter-defibrillator; LVEF, left ventricular ejection fraction; MRA, mineralocorticoid receptor antagonist; NT-proBNP, N-terminal pro-B-type natriuretic peptide; NYHA, New York Heart Association; SGLT2i, sodium–glucose cotransporter 2 inhibitor.

**Supplemental Table 2. Baseline characteristics for treatment allocation, stratified by sex**

|  | **Natriuresis guided therapy** | | | **Standard of care** | | |
| --- | --- | --- | --- | --- | --- | --- |
|  | **Men N=89** | **Women N=61** | **p.overall** | **Men N=83** | **Women N=77** | **p.overall** |
| **Demographics** |  |  |  |  |  |  |
| Age (in years) at time of screening | 73 [66;80] | 75 [66;84] | 0.215 | 72 [64;80] | 76 [65;83] | 0.273 |
| Race, Caucasian | 87 (98%) | 55 (93%) | 0.168 | 79 (95%) | 76 (100%) | 0.498 |
| **Physical examination** |  |  |  |  |  |  |
| BMI (kg/m2) | 29 (4) | 27 (6) | 0.238 | 27 (6) | 30 (8) | 0.090 |
| Systolic blood pressure (mmHg) | 130 (25) | 133 (29) | 0.401 | 133 (28) | 130 (25) | 0.501 |
| Diastolic blood pressure (mmHg) | 80 (16) | 79 (18) | 0.776 | 82 (20) | 80 (20) | 0.681 |
| Heart rate | 88 (25) | 94 (29) | 0.216 | 90 (26) | 103 (30) | 0.004 |
| NYHA class |  |  | 0.279 |  |  | 0.636 |
| Class II | 2 (2%) | 3 (5%) |  | 7 (8%) | 4 (5%) |  |
| Class III | 27 (30%) | 12 (20%) |  | 16 (19%) | 13 (17%) |  |
| Class IV | 60 (67%) | 46 (75%) |  | 60 (72%) | 60 (78%) |  |
| **HF characteristics** |  |  |  |  |  |  |
| LVEF (%) | 36 (14) | 40 (14) | 0.187 | 36 (13) | 40 (13) | 0.131 |
| HFpEF | 16 (24%) | 14 (29%) | 0.640 | 18 (13%) | 13 (26%) | 0.122 |
| Ischemic aetiology | 44 (49%) | 12 (20%) | <0.001 | 35 (42%) | 20 (26%) | 0.047 |
| New-onset HF | 36 (40%) | 30 (49%) | 0.373 | 36 (44%) | 33 (43%) | 1.000 |
| Time since HF diagnosis (months) | 10 [0;72] | 0 [0;54] | 0.614 | 6 [0;94] | 27 [0;74] | 0.937 |
| **Medical history** |  |  |  |  |  |  |
| Myocardial infraction | 36 (40%) | 12 (20%) | 0.012 | 32 (39%) | 20 (26%) | 0.126 |
| Hypertension | 59 (66%) | 34 (56%) | 0.256 | 51 (61%) | 51 (66%) | 0.642 |
| Atrial fibrillation | 48 (54%) | 36 (59%) | 0.654 | 48 (58%) | 42 (55%) | 0.796 |
| Diabetes | 31 (35%) | 21 (34%) | 1.000 | 37 (45%) | 29 (38%) | 0.467 |
| Depression | 4 (4%) | 1 (2%) | 0.649 | 7 (8%) | 5 (6%) | 0.869 |
| **Laboratory values** |  |  |  |  |  |  |
| Haemoglobin (mmol l^−1^) | 7.7 [6.9;8.7 | 7.7 [6.8;8.5] | 0.569 | 7.7 [6.8;8.6] | 7.9 [7.2;8.8] | 0.455 |
| Haematocrit (%) | 0.4 [0.3;0.4] | 0.4 [0.3;0.4] | 0.615 | 0.4 [0.3;0.4] | 0.4 [0.4;0.4] | 0.440 |
| Sodium (mmol/L) | 138 [134;140] | 136 [133;139] | 0.052 | 137 [134;140] | 137 [134;139] | 0.204 |
| Potassium (mmol l^−1^) | 4.5 [4.0;4.8] | 4.2 [4.0;4.4] | 0.014 | 4.4 [4.0;4.7] | 4.2 [3.9;4.6] | 0.190 |
| Creatinine (mmol/L) | 117 [93;163] | 88 [74;128] | <0.001 | 122 [95;181] | 94 [75;110] | <0.001 |
| eGFR (ml/min/1.73m2) - CKD EPI | 54 [35;72] | 55 [34;72] | 0.733 | 50 [32;70] | 55 [42;75] | 0.190 |
| NT-proBNP (ng/L) | 5816 [2515;9033] | 3860 [2652;7023] | 0.229 | 4995 [2788;11299] | 4931 [1975;8732] | 0.317 |
| **Treatment** |  |  |  |  |  |  |
| ACEi/ARB/ARNI | 50 (56%) | 32 (52%) | 0.777 | 47 (57%) | 40 (52%) | 0.664 |
| ACEi | 25 (28%) | 18 (30%) | 0.996 | 31 (37%) | 16 (21%) | 0.034 |
| ARB | 16 (18%) | 11 (18%) | 1.000 | 11 (13%) | 18 (23%) | 0.146 |
| ARNI | 9 (10%) | 3 (5%) | 0.361 | 5 (6%) | 6 (8%) | 0.897 |
| Beta-blockers | 62 (70%) | 38 (62%) | 0.445 | 60 (72%) | 55 (71%) | 1.000 |
| MRA | 32 (36%) | 21 (34%) | 0.985 | 23 (28%) | 31 (40%) | 0.131 |
| SGLT2i | 4 (4%) | 4 (7%) | 0.716 | 6 (7%) | 6 (8%) | 1.000 |
| Home loop diuretic | 49 (55%) | 35 (57%) | 0.909 | 51 (61%) | 42 (55%) | 0.469 |
| Home loop diuretic dose (mg of bumetanide equivalents) | 2 [1;4] | 2 [1;4] | 0.608 | 2 [1;4] | 2 [1;4] | 0.248 |
| ICD | 22 (25%) | 11 (18%) | 0.441 | 23 (28%) | 12 (16%) | 0.096 |
| CRT | 12 (13%) | 5 (8%) | 0.459 | 8 (10%) | 4 (5%) | 0.444 |
| **Congestion** |  |  |  |  |  |  |
| Rales |  |  | 0.965 |  |  | 0.123 |
| Absent | 23 (26%) | 18 (30%) |  | 21 (27%) | 23 (31%) |  |
| < 1/3 lungs | 41 (47%) | 26 (43%) |  | 40 (51%) | 26 (35%) |  |
| 1/3 to 2/3 lungs | 17 (19%) | 12 (20%) |  | 13 (16%) | 22 (30%) |  |
| >2/3 lungs | 7 (8%) | 5 (8%) |  | 5 (6%) | 3 (4%) |  |
| Edema |  |  | 0.031 |  |  | 0.725 |
| Absent/trace | 23 (27%) | 23 (38%) |  | 28 (35%) | 23 (32%) |  |
| Slight | 14 (17%) | 9 (15%) |  | 13 (16%) | 8 (11%) |  |
| Moderate | 28 (33%) | 8 (13%) |  | 17 (21%) | 18 (25%) |  |
| Marked | 19 (23%) | 21 (34%) |  | 23 (28%) | 24 (33%) |  |
| JVP |  |  | 0.720 |  |  | 0.815 |
| Elevated | 21 (34%) | 20 (40%) |  | 26 (39%) | 23 (39%) |  |
| Normal | 9 (15%) | 8 (16%) |  | 15 (23%) | 11 (19%) |  |
| Unknown | 32 (52%) | 22 (44%) |  | 25 (38%) | 25 (42%) |  |
| Orthopnoea | 49 (63%) | 36 (73%) | 0.295 | 49 (71%) | 40 (74%) | 0.862 |

ACEi, angiotensin-converting enzyme inhibitor; ARB, angiotensin receptor blocker; ARNI, angiotensin receptor–neprilysin inhibitor; BMI, body mass index; CKD, chronic kidney disease; CRT, cardiac resynchronization therapy; eGFR, estimated glomerular filtration rate; HF, heart failure; HFrEF, heart failure with reserved ejection fraction; HFpEF, heart failure with preserved ejection fraction; ICD, implantable cardioverter-defibrillator; LVEF, left ventricular ejection fraction; MRA, mineralocorticoid receptor antagonist; NT-proBNP, N-terminal pro-B-type natriuretic peptide; NYHA, New York Heart Association; SGLT2i, sodium–glucose cotransporter 2 inhibitor.

**Supplemental Table 3. Safety endpoints according to treatment group and sex**

|  | **Men** | | | **Women** | | |
| --- | --- | --- | --- | --- | --- | --- |
|  | **Natriuresis guided therapy N=89** | **Standard of care N=83** | **p-value** | **Natriuresis guided therapy N=61** | **Standard of care N=77** | **p-value** |
| Adverse event | 51 (57%) | 55 (66%) | 0.293 | 35 (57%) | 41 (53%) | 0.755 |
| Serious adverse event | 37 (42%) | 37 (45%) | 0.807 | 23 (38%) | 33 (43%) | 0.662 |

**Supplemental Table 4. Cox regression analysis for good/poor natriuresis response with and without WRF to the 180-day primary endpoints**

|  | **Men n=139** | **Women n=112** | **P for sex interaction** |
| --- | --- | --- | --- |
| **Good/poor natriuresis (24h) with and without WRF** | | | |
| **Good natriuresis without WRF**, n (%) | 47 (34%) Reference | 30 (27%) Reference | - |
| **Good natriuresis with WRF** |  |  |  |
| n (%) | 34 (24%) | 19 (17%) |  |
| Unadjusted HR (95%CI) | 0.97 (0.41-2.27) p=0.943 | 0.99 (0.24-4.16) p=0.994 | 0.977 |
| Adjusted HR (95%CI) | 1.35 (0.55-3.3) p=0.513 | 1.20 (0.26-5.61) p=0.816 | 0.992 |
| **Poor natriuresis without WRF** |  |  |  |
| n (%) | 39 (28%) | 42 (38%) |  |
| Unadjusted HR (95%CI) | **2.75 (1.38-5.47) p=0.004** | 1.35 (0.45-4.02) p=0.594 | 0.272 |
| Adjusted HR (95%CI) | **3.31 (1.61-6.79) p=0.001** | 1.19 (0.36-3.93) p=0.77 | 0.134 |
| **Poor natriuresis with WRF** |  |  |  |
| n (%) | 19 (14%) | 21 (19%) |  |
| Unadjusted HR (95%CI) | 2.15 (0.92-5.02) p=0.079 | 2.69 (0.88-8.22) p=0.083 | 0.756 |
| Adjusted HR (95%CI) | 2.45 (0.99-6.05) p=0.053 | 1.72 (0.47-6.32) p=0.415 | 0.744 |

**Supplemental Figure 1. Total loop diuretic doses over time (Mean and 95%CI)**

Mean values are shown with dots, and the bars represent 95% CI. P-value were calculated in linear mixed-effect models.

Men (natriuresis-guided treatment versus SOC): ANOVA main p-value for difference between the models (p<0.001)

Women (natriuresis-guided treatment versus SOC): ANOVA main p-value for difference between the models (p<0.001)
p for sex interaction=0.504
